# Besondere Aspekte von Patienten mit va-ECMO auf dem Weg zur Organspende

**DOI:** 10.1007/s00101-025-01550-5

**Published:** 2025-06-18

**Authors:** Esther Tautz, Johann Lambeck, Klaus Michael Lücking, Dominik Bettinger, Michael Zinner, Alexander Supady, Guido Michels, Dawid L. Staudacher

**Affiliations:** 1 Interdisziplinäre Medizinische Intensivtherapie (IMIT), Universitätsklinikum Freiburg, Medizinische Fakultät, Universität Freiburg, Hugstetter Str. 55, 79106 Freiburg, Deutschland; 2 Klinik für Neurologie und Neurophysiologie, Universitätsklinikum Freiburg, Breisacherstr. 64, 79106 Freiburg, Deutschland; 3 Stabsstelle Organspende, Universitätsklinikum Freiburg, Freiburg im Breisgau, Deutschland; 4 Klinik für Innere Medizin II, Universitätsklinikum Freiburg, Medizinische Fakultät, Albert-Ludwigs-Universität Freiburg, Hugstetter Str. 55, D-79106 Freiburg, Deutschland; 5 Notfallzentrum, Krankenhaus der Barmherzigen Brüder Trier, Medizincampus Trier der Universitätsmedizin Mainz, Trier, Deutschland

**Keywords:** Organprotektive Therapie, Intensivmedizin, Irreversibler Hirnfunktionsausfall, Venoarterielle Membranoxygenierung, Multiorganversagen, Donor management, Intensive care, Brain death/death by neurologic criteria, Veno-arterial extracorporeal membrane oxygenation, Multiorgan failure

## Abstract

Die Behandlung von potenziellen Organspendern an einer venoarteriellen extrakorporalen Membranoxygenierung (va-ECMO) ist komplex und ressourcenintensiv, sowohl technisch durch die ECMO selbst als auch durch die Grunderkrankung. Zudem können im Rahmen eines irreversiblen Hirnfunktionsausfalls (IHA) profunde pathophysiologische Veränderungen auftreten, die insbesondere kardiorespiratorische, immunologische und endokrinologische Störungen bewirken. Sowohl bezüglich der Feststellung des IHA als auch bezüglich kardiorespiratorischer, gastroenterologischer, nephrologischer und gerinnungsbezogener Aspekte sind spezifische Kenntnisse erforderlich. Gerade angesichts des Mangels an Spenderorganen sollte auch diesem Patientenkollektiv der Wunsch einer Organspende nicht verwehrt werden, falls es im Rahmen einer deletären Hirnschädigung unter laufender ECMO zu einem IHA kommen sollte. Ziel dieses Übersichtsartikels ist, eine Zusammenfassung besonderer Aspekte in der Betreuung potenzieller Organspender an der ECMO zu geben.

## Abstract (en)

The treatment of potential organ donors on veno-arterial extracorporeal membrane oxygenation (va-ECMO) is complex and resource-intensive due to the challenges of ECMO support itself and due to the severity of the underlying disease, typically associated with profound shock. This shock can lead to multiorgan failure, which can negatively impact the suitability of potential organs for transplantation; however, ECMO offers a chance for recovery of acutely impaired organs, as adequate perfusion is provided. In addition, profound pathophysiological changes can occur in the context of brain death/death by neurologic criteria (BD/DNC) which, in particular, can cause cardiorespiratory, immunological and endocrinological disorders. Specific knowledge is required concerning the diagnosis of BD/DNC as well as cardiorespiratory, gastroenterological, nephrological and coagulation-related aspects. Particularly in view of the shortage of donor organs, this group of patients should not be denied the wish for organ donation if BD/DNC occurs in the context of severe brain injury under ongoing ECMO support. The aim of this review article is to provide an overview of special aspects in the care of potential organ donors on ECMO.

## Einleitung

Die Behandlung von potenziellen Organspendern an einem extrakorporalen mechanischen Unterstützungssystem wie einer extrakorporalen Membranoxygenierung (ECMO) ist komplex. Ziel dieser Arbeit ist, einen Überblick über besondere Aspekte und Herausforderungen in der Betreuung dieser Patientengruppe zu geben.

## Hintergrund

Mechanische Unterstützungssysteme kommen zunehmend zur Verbesserung der Hämodynamik („veno-arterial extracorporeal membrane oxygenation“, va-ECMO) und des Gasaustausches („veno-venous extracorporeal membrane oxygenation“, vv-ECMO) zur Anwendung [37].

Ein besonderes Anwendungsgebiet der va-ECMO ist der Einsatz bei Patienten im therapierefraktären Kreislaufstillstand, die sog. extrakorporale Reanimation („extracorporeal cardiopulmonary resuscitation“, ECPR). Die ECPR stellt hierbei ein ressourcenintensives Verfahren dar [29]. Dennoch überleben unter optimalen Bedingungen in selektierten Kohorten nur ca. 20–43 % der Patienten [35, 36, 42]. Ein Grund für die hohe Mortalität ist die oft lange Reanimationsdauer mit entsprechend protrahierter Hypoxiezeit [38]. So kommt es zu einer steigenden Anzahl an Patienten an der va-ECMO mit hypoxischen Hirnschädigungen [44]. Das Ausmaß der Schädigung kann von leichten Defiziten bis hin zu einer neurologisch infausten Prognose und zum irreversiblen Hirnfunktionsausfall (IHA) reichen [3, 35]. Ein weiterer Grund für einen IHA kann eine massive zerebrale Blutung unter bereits laufender ECMO sein [25, 39, 45]. In beiden Fällen kommen die Patienten mit IHA für eine postmortale Organspende in Frage.

Ein weiteres Szenario ist eine therapierefraktäre hämodynamische Instabilität im Rahmen eines IHA, da dieser profunde pathophysiologische Veränderungen, die insbesondere kardiorespiratorische, immunologische und endokrinologische Störungen auslösen, bewirkt [2, 28]. Da potenzielle Organspender trotz dezidierten intensivmedizinischen Managements einen schweren Schock bis hin zum Kreislaufstillstand erleiden können [23, 28], kann in dieser Situation eine va-ECMO als Behandlungsoption mit dem Ziel der Ermöglichung einer Organspende diskutiert werden [8].

Häufig entwickeln Patienten mit va-ECMO aufgrund eines prolongierten Schocks ein Multiorganversagen [15, 17]. Dies hat einen negativen Einfluss auf die Eignung der Organe für eine Organspende. Andererseits besteht die Chance einer Erholung akut geschädigter Organe durch die mittels va-ECMO gesicherte Perfusion, beispielsweise im Fall einer hypoxischen Hepatitis oder eines akuten Nierenversagens („acute kidney injury“, AKI) [4, 19], insbesondere gegenüber einer ansonsten oft erforderlichen hochdosierten Katecholamintherapie [10].

In Anbetracht des Mangels an Spenderorganen ist es notwendig, die Zahl der Organspenden bzw. der Spenderorgane zu erhöhen. Hierzu ist eine zielgerichtete intensivmedizinische Therapie im Sinne der Organprotektion unverzichtbar [9, 21, 23, 26, 28]. Ziel der organprotektiven Intensivtherapie ist der Erhalt der Homöostase, welche u. a. durch die oben diskutierte deletäre Hirnschädigung und zerebrale Einklemmung gefährdet ist. Akut geschädigte Organe können sich unter einer optimalen organerhaltenden Intensivtherapie funktionell erholen und konsekutiv als Spenderorgane in Betracht kommen.

Die Ergänzung einer Intensivtherapie zur Organprotektion mittels eines mechanischen Unterstützungssystems ist auf vielen Ebenen (medizinisch, ethisch, rechtlich, organisatorisch) komplex. Eine Therapieeskalation sollte sowohl im intensivmedizinischen Team als auch mit den Angehörigen konsentiert werden. Hilfestellung in diesen Abwägungsprozessen gibt eine interdisziplinär erarbeitete Empfehlung der Deutschen Interdisziplinären Vereinigung für Intensiv- und Notfallmedizin [30]. Die Richtlinie zur Spendererkennung der Bundesärztekammer (BÄK) greift diese Thematik ebenfalls auf und erachtet Maßnahmen zur Wiederherstellung der Herz-Kreislauf-Funktion als angemessen, wenn diese dem Patientenwillen entsprechen [1].

Auf Grund der komplexen Therapie von ECMO-Patienten mit IHA soll im Folgenden das intensivmedizinische Management im Kontext einer Organspende dargestellt werden.

## Spezielle Aspekte der IHA-Diagnostik an der va-ECMO

Bei der Hirntodfeststellung (IHA-Diagnostik) von Patienten an der va-ECMO gibt es spezielle Aspekte, die berücksichtigt werden müssen. Dies betrifft alle 3 Stufen des Algorithmus der entsprechenden Richtlinie der BÄK (Prüfung der Voraussetzungen, klinische Symptome, Nachweis der Irreversibilität) [1].

Auf Stufe 1 sollte bedacht werden, dass viele Patienten dieses Kollektivs über längere Zeit tief sediert und gekühlt wurden und die Pharmakokinetik und -dynamik verändert sein können (z. B. Verlängerung der Halbwertszeit von Analgetika und Sedativa) [7]. Da diese Medikamente die Wertung der bei der klinischen Untersuchung erhobenen Befunde (Stufe 2) beeinflussen können, ist hier im Zweifel die Bestimmung der Plasmakonzentration empfehlenswert, jedoch nicht in der aktuellen Richtlinie der Bundesärztekammer gefordert [1]. Da es für die Beurteilung medikamentöser Einflüsse auf bestimmte Befunde im Kontext der vorliegenden Hirnschädigungen keine gesicherten Konzentrations-Wirkungs-Beziehungen gibt, muss eine mögliche Relevanz der gemessenen Konzentrationen beispielsweise von Benzodiazepinen oder Opiaten gemeinsam durch in die IHA-Diagnostik involvierte Neurologen, Intensivmediziner und ggf. Pharmakologen oder Toxikologen bewertet werden [1]. Dies kann zu zeitlichen Verzögerungen führen, da nicht jedes Labor über die erforderliche toxikologische Analytik verfügt und Material ggf. in für rechtsmedizinische Untersuchungen akkreditierte Labore gesandt werden muss.

Auf Stufe 2 ist insbesondere die Durchführung des Apnoetestes erwähnenswert. Ein PEEP-Verlust durch eine Diskonnektion vom Beatmungsgerät sollte bei diesen potenziell kardiopulmonal instabilen Patienten vermieden werden [24]. Der Nachweis eines erhöhten CO_2_ im Rahmen des Apnoetestes kann durch das Vorhandensein einer Wasserscheide (auch differenzielle Zirkulation oder Harlekin-Phänomen genannt) [33] kompliziert werden, wenn ein patienteneigener kardialer Auswurf besteht. Diese Wasserscheide kann dazu führen, dass das Gehirn antegrad mit Blut aus dem Herzen des Patienten, der Körper distal der Wasserscheide retrograd mit Blut aus der va-ECMO perfundiert wird. Die jeweiligen Partialdrücke für O_2_ und CO_2_ sind dabei nicht notwendigerweise gleich. Eine von uns propagierte Methode bei Patienten mit kardialem Auswurf und anzunehmender Wasserscheide ist eine bilaterale Blutgasanalyse aus beiden Armen. Die Steuerung der va-ECMO-Einstellungen sollte unter Mitwirkung des intensivmedizinischen Behandlungsteams erfolgen (konkrete Durchführung: FiO_2_ am Beatmungsgerät und der ECMO auf 100 % einstellen, Sweep-Gasfluss auf 0,5–1,0 l/min reduzieren, Blutfluss unverändert belassen [40]), um so eine valide Aussage bezüglich eines fehlenden Atemantriebs im Rahmen des IHA zu erhalten.

Bei der Überprüfung der Irreversibilität des klinischen Befundes (Stufe 3) ist zu beachten, dass in der aktuell gültigen Richtlinie der BÄK [1] die Anwendung sämtlicher Perfusionsverfahren (Doppler‑/Duplexsonographie, CT-Angiographie, SPECT) bei Patienten an der va-ECMO für unzulässig erklärt wurde. Auch wenn unter Beachtung bestimmter Voraussetzungen belastbare Ergebnisse in den Perfusionsverfahren an der va-ECMO gewonnen werden könnten, dürfen diese somit nicht in die IHA-Diagnostik einbezogen werden. Anders ist dies bei der vv-ECMO; hier sind alle Perfusionsverfahren zulässig.

Für Patienten ab Beginn des 3. Lebensjahres und eine vollständig durchführbare und verwertbare klinische Untersuchung vorausgesetzt, kann die IHA-Diagnostik entweder durch eine 2‑zeitige klinische Untersuchung (je nach Schädigungsmuster im Abstand von 12 bzw. 72 h), die ergänzende Durchführung einer Elektroenzephalographie (EEG) bzw. (je nach Schädigungsmuster) von somatosensibel evozierten Potenzialen (SEP) oder den frühen akustisch evozierten Potenzialen (FAEP) vervollständigt werden. Da die meisten Patienten dieses Kollektivs isoliert oder in Kombination eine sekundäre Hirnschädigung (z. B. hypoxischer Hirnschaden nach CPR) aufweisen, müsste hier eine Wartezeit von 72 h für die zweite klinische Untersuchung einkalkuliert werden. Dies ist aufgrund der typischerweise vorliegenden kardiopulmonalen Instabilität meist nicht realistisch. Die EEG wiederum kann auf Grund der häufig hochdosiert applizierten Sedativa bei verzögerter Metabolisierung und mithin hochtherapeutischen oder toxischen Spiegeln erschwert sein. Im Fall einer isoliert oder in Kombination vorliegenden infratentoriellen Schädigung (z. B. intrazerebrale Blutung oder Ischämie der hinteren Schädelgrube) dürfen SEP und FAEP nicht angewendet werden. Dieses Dilemma kann dazu führen, dass eine richtliniengemäße IHA-Diagnostik und konsekutiv auch eine Organspende nicht möglich sind.

## Blutungskomplikationen

Der Einsatz extrakorporaler Verfahren geht mit einem signifikant erhöhten Risiko für Blutungskomplikationen einher. Die Blutungsrate unter va-ECMO ist höher als unter vv-ECMO, die Inzidenz jedoch auf Grund der typischerweise längeren Laufzeit der vv-ECMO vergleichbar. Mindestens jeder dritte Patient entwickelt eine potenziell lebensbedrohliche Blutungskomplikation während der ECMO-Unterstützung [31]. Intrazerebrale Blutungen werden je nach untersuchtem Kollektiv bei bis zu jedem zehnten Patienten berichtet [25]. Gründe für Blutungen sind neben den großlumigen Einstichstellen auch der Verbrauch von Gerinnungsfaktoren [18], eine reduzierte Synthese von pro- und antikoagulatorischen Faktoren im Rahmen einer Leberfunktionsstörung sowie die erforderliche Antikoagulation [5]. Eine zu liberale Substitution ist jedoch ressourcenaufwendig und kann zu thromboembolischen Komplikationen führen.

## Hepatologische und gastroenterologische Probleme

Patienten nach Kreislaufstillstand mit der Notwendigkeit einer Kreislaufunterstützung mittels va-ECMO haben ein hohes Risiko, gastrointestinale und hepatologische Komplikationen zu erleiden. Diese Komplikationen lassen sich v. a. auf eine stattgehabte Minderperfusion des Darms und der Leber im Rahmen des Schocks zurückführen.

Eine protrahierte Minderperfusion der Leber kann zu einer hypoxischen Hepatitis führen. Pathophysiologisch spielen neben der Ischämie auch eine systemische Hypoxie, eine eingeschränkte Sauerstoffextraktion in den Hepatozyten und eine venöse Stauung eine Rolle [16]. Laborchemisch zeigt sich die hypoxische Hepatitis durch einen massiven Anstieg der Transaminasen innerhalb der ersten Stunden nach Kreislaufstillstand. Die Aspartataminotransferase (AST) ist in dieser Initialphase signifikant höher als die Alaninaminotransferase (ALT). Nach Wiederherstellung eines suffizienten Kreislaufs kommt es in der Regel zum Abfall der Transaminasen, wobei die AST auch deutlich schneller abfällt [11, 14]. In 36 % der Patienten mit einer hypoxischen Hepatitis steigt das Bilirubin 2 bis 6 Tage nach Abfall der Transaminasen an [16]. Zusammenfassend besteht die Therapie der hypoxischen Hepatitis aus einer schnellen Wiederherstellung adäquater Kreislaufverhältnisse [16]. Ergänzend sollten alternative Ursachen für eine Leberschädigung ausgeschlossen werden. Eine Kreislaufunterstützung mittels va-ECMO erscheint pathophysiologisch zielführend, wobei prospektive Studien fehlen.

Das Vorliegen einer hypoxischen Hepatitis kann ein Hinweis darauf sein, dass auch eine Minderperfusion des Darms im Sinne einer nichtokklusiven Mesenterialischämie (NOMI) vorliegen könnte. Teilweise ergeben sich in der initial nach der CPR durchgeführten Computertomographie (CT) bereits Hinweise darauf. Hierbei muss jedoch bedacht werden, dass die CT-morphologischen Zeichen einer NOMI sehr unspezifisch sind [20]. Da eine unerkannte Ischämie des Dünn- und Dickdarms eine therapielimitierende Sepsis verursachen kann, ist die frühzeitige Detektion essenziell. In unserem Zentrum hat sich das Konzept einer früh-elektiven Screeningkoloskopie bei Patienten nach ECPR als effektives und sicheres Verfahren herausgestellt. Hierbei zeigt sich, dass die Rate an klinisch unerkannten Kolonischämien mit bis zu 40 % signifikant höher ist als in der Literatur angegeben [20, 41]. Mittels früh-elektiver Koloskopie kann eine Darmischämie frühzeitig erkannt werden, um die weiteren vorbereitenden Untersuchungen für die Organspende zu beschleunigen.

## Kardiopulmonale Probleme

Häufig liegt einem therapierefraktären Kreislaufstillstand mit folgender ECPR eine kardiale Ursache zu Grunde. Somit ist nicht verwunderlich, dass die Rate an gespendeten Herzen in dieser Patientengruppe signifikant niedriger ist als bei Patienten ohne ECPR [4, 43]. Auch die Lungen kommen nach einer ECPR aufgrund von Schädigungen durch Thoraxkompressionen oder eine Aspiration im Rahmen der prolongierten Reanimation selten für eine Organspende in Frage [3, 35]. Andererseits ermöglicht die ECMO eine niedrig-invasive Beatmung, so dass respiratorassoziierte Schädigungen der Lunge vermieden werden können [32]. Durch die ECMO wird zudem die Sauerstoffversorgung anderer, für eine Organspende geeignete Organe sichergestellt [4, 19].

Eine gefürchtete Komplikation bei Patienten mit hochgradig reduzierter linksventrikulärer Funktion an einer va-ECMO ist die Blutstase im linken Ventrikel. Die Stase kann zur Thrombosierung des Herzens und zur schweren pulmonalvenösen Kongestion mit Lungenversagen führen [27]. Ursächlich ist das Unvermögen des schwer geschädigten linken Ventrikels, Blut gegen die durch die va-ECMO retrograd gefüllte Aorta und die somit hohe Nachlast auszuwerfen. In diesem Falle kommen sog. linksventrikuläre Venting-Verfahren z. B. mittels Mikroaxialpumpe zum Einsatz [34]. Die Kombination von zwei extrakorporalen Unterstützungssystemen erfordert eine hohe technische und klinische Expertise. Zudem ist stets zu diskutieren, wie hoch die Therapieinvasivität in Abwägung mit dem Patientenwillen auf dem Weg zu einer Organspende sein darf [30]. Hier fehlen klare Richtlinien, was einen hohen Anspruch an Umsichtigkeit an das Behandlungsteam in der Behandlung dieser schwerstkranken Patienten sowie in der Kommunikation mit den Zugehörigen stellt.

## Nephrologische Aspekte

Nach Minderperfusion im Schockgeschehen oder Kreislaufstillstand besteht ein hohes Risiko für ein AKI. Insbesondere nach einer ECPR findet sich diesbezüglich eine hohe Inzidenz [12]. Mittels va-ECMO lässt sich eine suffiziente Perfusion der Nieren sichern, so dass trotz Ischämie-Reperfusionsschaden die Chance auf eine Erholung der Nierenfunktion besteht [19]. Nach einer ECPR sind die Nieren die am häufigsten zu transplantierenden Organe [3, 6, 35]. Bezüglich der ECMO-Therapie gilt es zu beachten, dass die Nieren bei einem Sweep-Gasfluss mit hoher Sauerstoffkonzentration oxidativem Stress ausgesetzt sein können, was wiederum das Risiko eines AKI erhöht [13]. Besonders bei Patienten mit einer Wasserscheide distal der supraaortalen Gefäße sollte die Durchführung einer Blutgasanalyse aus der ECMO (Postoxygenatorblutgasanalyse) erwogen und die Sauerstofffraktion des Sweep-Gasflusses entsprechend angepasst werden.

**Abb. 1:**
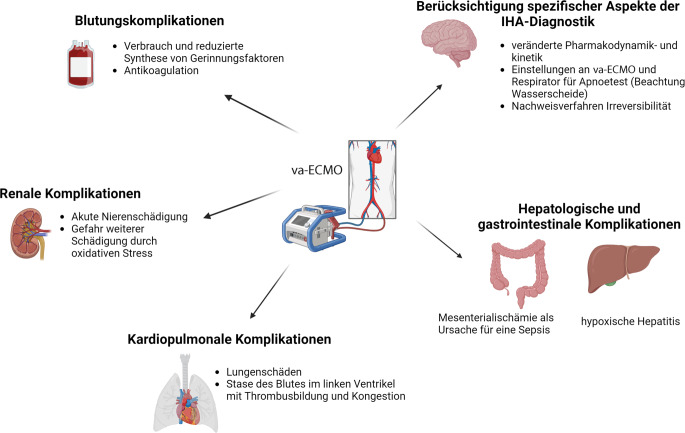
Organsystembezogene Aspekte (erstellt mit BioRender)

In Abb. 1 fassen wir organsystembezogen wesentliche Aspekte zusammen.

## Outcome

Es gibt wenige Daten zur Organspende nach einer ECPR. In der Prague-OHCA-Studie, der größten randomisierten Studie zu ECPR, starben 75/256 (29 %) der Teilnehmer präklinisch oder innerhalb einer Stunde nach Erreichen der Klinik und konnten daher nicht für eine Organspende evaluiert werden. Während des Klinikaufenthalts starben 107 der 256 Studienteilnehmer (43 %) und kamen daher potenziell für eine Organspende in Betracht. 24 (9,4 % aller Studienteilnehmer) wurden als Organspender evaluiert und bei 15 Patienten mit IHA (13 nach ECPR, 2 aus der Kontrollgruppe; 5,9 % aller Studienteilnehmer) konnte letztlich eine Organspende realisiert werden [35].

In der INCEPTION-Studie wurde bei 56/70 Patienten in der ECPR-Gruppe die Therapie auf der Intensivstation eingestellt. Bei 29 dieser Patienten waren eine ungünstige neurologische Prognose oder fehlende Therapieoptionen der Grund für die Therapieeinstellung [36]. Diese Patienten (d. h. 41 % der Patienten in der ECPR-Gruppe) könnten potenzielle Organspender sein.

Im Rahmen des ECPR-Programmes des Minnesota Resuscitation Consortium konnten sowohl die Anzahl als auch der Anteil der Organspenden nach ECPR (2023: 71 %) signifikant gesteigert werden, wie eine retrospektive Analyse zeigte [22].

Trotz der Komplexität dieses schwer kranken Patientenkollektivs lassen sich gute Transplantationsergebnisse erzielen [6]. Eine retrospektive Kohortenstudie zeigte ein schlechteres Transplantatüberleben für Lungen, die nach ECPR gespendet wurden. Für die anderen transplantablen Organe waren die Ergebnisse gleich gut [43]. In einer weiteren Studie fand sich im Vergleich von Organspendern an der va-ECMO zu Patienten ohne va-ECMO kein signifikanter Unterschied zwischen Transplantatüberleben und -funktion der Nieren nach einem Jahr sowie dem Einjahresüberleben von lebertransplantierten Patienten [4].

Auch eine Analyse des Prague OHCA Trial sowie der Daten aus Minnesota ergab ein exzellentes Ergebnis mit guter Funktion aller transplantierten Organe [22, 35].

**Abb. 2:**
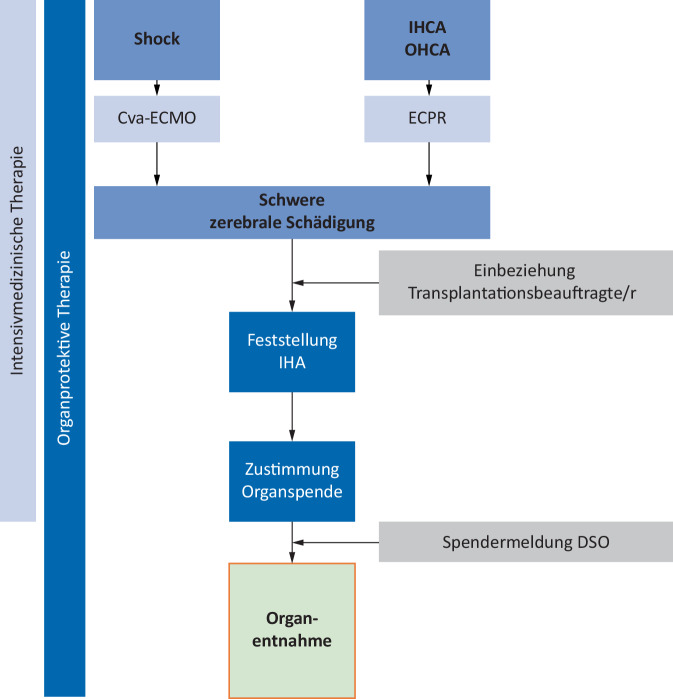
Flowchart. *OHCA* „out-of-hospital cardiac arrest“, *IHCA* „in-hospital cardiac arrest“, *ECPR* „extracorporeal cardiopulmonary resuscitation“, *va/vv-ECMO* „veno-arterial/veno-venous extracorporeal membrane oxygenation“, *IHA* irreversibler Hirnfunktionsausfall, *DSO* Deutsche Stiftung Organtransplantation

Im Flowchart der Abb. 2 fassen wir unser Vorgehen bei ECMO-Patienten, die für eine Organspende evaluiert werden, zusammen.

## Fazit

Die Therapie von potenziellen Organspendern an einem mechanischen Unterstützungssystem ist komplex. Besondere Aspekte betreffen insbesondere die IHA-Diagnostik, das kardiorespiratorische sowie Gerinnungsmanagement sowie die frühzeitige Erkennung einer hypoxischen Hepatitis oder Mesenterialischämie. Die Betreuung der Patienten ist äußerst ressourcenintensiv und der Ausgang ungewiss. Sollten ein Abbruch der Therapie erforderlich und Bemühungen, eine Organspende zu ermöglichen, letztlich frustran sein, kann dies eine zusätzliche Belastung für das Team und die Zugehörigen darstellen. Trotzdem sollte in allen Fällen mit neurologisch infauster Prognose der Patientenwille hinsichtlich einer Organspende eruiert werden. In unserer Erfahrung besteht selbst bei komplex erkrankten Patienten an der va-ECMO durch eine strukturierte interdisziplinäre Zusammenarbeit eine realistische Chance, dem Patientenwunsch einer Organspende zu entsprechen und ein gutes Transplantationsergebnis zu erzielen.
